# Lysolipid containing liposomes for transendothelial drug delivery

**DOI:** 10.1186/1756-0500-5-179

**Published:** 2012-04-10

**Authors:** Tilen Koklic, Janez trancar

**Affiliations:** 1 Laboratory of Biophysics (http://lbf.ijs.si), Condensed Matter Physics F5, Joef Stefan Institute, Jamova 39, SI-1000, Ljubljana, Slovenia; 2 Center of Excellence Namaste, Advanced Bio Materials, Jamova 39, SI-1000, Ljubljana, Slovenia

## Abstract

**Background:**

Designing efficient 'vectors', to deliver therapeutics across endothelial barriers, in a controlled manner, remains one of the key goals of drug development. Recently, transcytosis of liposome encapsulated fluorescence marker calcein across a tight cell barrier was studied. The most efficient liposomes were found to be liposomes containing sufficient amount of alkyl phospholipid (APL) perifosine. APLs have similar structure as lysophosphatidyl choline (LPC), since APLs were synthesized as metabolically stable analogues of LPC, which increases endothelial permeability directly by inducing endothelial cell contraction, resulting in formation of gaps between endothelial cells. Since one of the unique properties of lysolipid, containing liposomal formulations is dynamic equilibrium of lysolipids, which are distributed among liposomes, micelles, and free form, such liposomes represent a reservoir of free lysolipids. On the other hand lysolipid containing liposomes also represent a reservoir of an encapsulated hydrophilic drug.

**Presentation of the hypothesis:**

We hypothesize that free lysolipids, with highest concentration in vicinity of drug carrying liposomes, compromise endothelial integrity, primarily where concentrations of liposomes is the highest, in a similar manner as LPC, by formation of gaps between endothelial cells. Liposome encapsulated drug, which leaks from liposomes, due to liposome destabilization, caused by lysolipid depletion, can therefore be efficiently transported across the locally compromised endothelial barrier.

**Testing the hypothesis:**

This hypothesis could be verified: by measuring binding of perifosine and other lysolipids to albumin and to lysophospholipid receptor (LPL-R) group; formation of stress fibers and subsequent cell contraction; activation of RhoA, and endothelial barrier dysfunction; by a synthesis of other LPC analogues with high critical micellar concentration and measuring their effect on transendothelial permeability in presence and absence of albumin.

**Implications of the hypothesis:**

We propose that lysolipid containing liposomal formulations might be used as nonspecific transendothelial transport vector, since leakage of liposome encapsulated active drug occurs simultaneously with the release of the lysolipids. The concentration of the active drug is therefore expected to be the highest at the site of compromised endothelial barrier. By appropriate choice of the lysolipids an endothelial barrier would stay open only for a short time. Use of such liposomes would potentially maximize the delivery of the drug while limiting the passage of toxic substances and pathogens across the endothelial barrier. Combining lysolipid containing liposomes with superparamagnetic iron oxide nanoparticles or a targeting ligand might be required to efficiently localize drug delivery to a disease affected tissue and to avoid endothelial disruption over the entire body.

## Background

### Transendothelial delivery of hydrophilic drugs

Transcytosis holds a great potential for drug delivery across different endothelial barriers. Designing efficient 'vectors' (antibodies, protein carriers, viruses, nanoparticles) to deliver therapeutics, especially to the disease-affected brain tissue, in a controlled and non-invasive manner remains one of the key goals of drug development [1]. Careful regulation of material exchange into and out of the brain is essential for the survival of neurons, which do not have a significant capacity to regenerate. This transport is regulated by the bloodbrain barrier (BBB), a dynamic interface between the blood and the brain formed by endothelial cells of the brain capillaries. However, it also very efficiently prevents the brain uptake of most therapeutically active compounds. Because of this, many diseases of central nervous system (CNS), such as Alzheimers disease, are undertreated. As a result, various strategies have been developed to improve the access of drugs to the brain parenchyma at therapeutically necessary concentrations to effectively manage diseases [2,3]. Various drug delivery systems such as: liposomes, surfactant coated polymeric nanoparticles, solid lipid nanoparticles [4], microspheres, nanogels, and bionanocapsules were tested for delivery of drugs to tumors of the CNS with different efficiancies [5][8]. Even though transcytosis is often thought to be a selective process, endothelial cells of microvasculature move macromolecular cargo rather nonselectively within the fluid phase of the transport vesicle or by absorption to the vesicle membrane [9]. Using vectors promoting transcytosis in such nonspecific manner can be more widely applied, especially in combination with nanoparticles or liposomes, into which large amounts of a drug can be incorporated [10]. Liposomes seem to be a promising delivery system, which enable high cellular uptake and efficient transcytosis across cellular barriers including the BBB, as their composition can be easily adjusted according to the properties of targeted cells and tissues [11]. It is expected that by a proper choice of liposome composition an efficient transcytosis of liposome entrapped drugs across the cellular barrier could be achieved [6]. Since the transport of such liposomes throughout the body cannot be controlled, they could be in principle produced with superparamagnetic iron oxide nanoparticles (SPIONs) or a targeting ligand in order to achieve their accumulation in desired tissue. SPIONs can be concentrated at a particular point of the body using external magnetic field [12].

### Recent research on perifosine liposomal formulations and transcellular delivery

**Figure 1:**
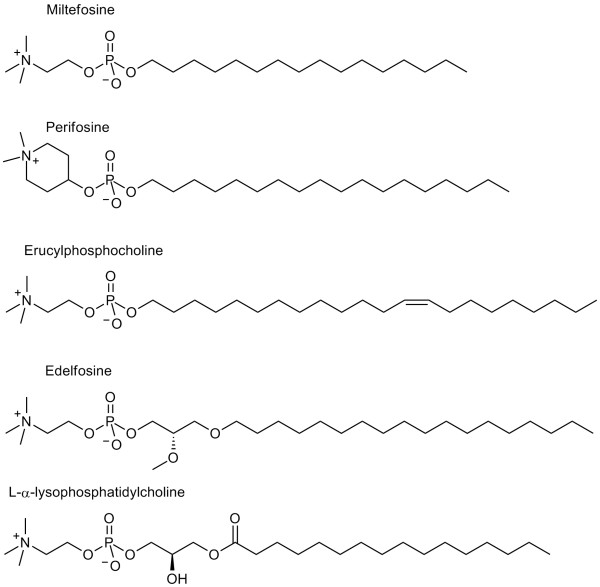
Structural formula of pharmaceutically tested alkylphospholipids and lysophosphatidylcholine.

In recent work by Orthman et al. [6] the effect of liposome bilayer properties on cellular uptake and transmembrane transport of the encapsulated hydrophilic marker calcein through a barrier formed by epithelial Madin-Darby canine kidney (MDCK) cells was investigated. A positive correlation between membrane fluidity in the upper part of the membrane bilayer and transcytosis was found [6]. Similarly, it has been suggested that also polyunsaturated fatty acids influence transendothelial transport of cortisol across an MDCK barrier by inducing changes in membrane fluidity and somehow affecting tight junction integrity [13]. Later analysis of the data by Orthman et al. (manuscript in preparation) revealed that among all liposome components the most pronounced correlation between any liposome component and transendothelial calcein delivery was found for an alkyl phospholipid (1,1-dimethylpiperidin 1-ium-4-yl) octadecyl phosphate (perifosine) concentration (Figure 1), namely the transendothelial delivery increased abruptly, in a nonlinear fashion, for liposomes containing more than 1:1 ratio of perifosine to cholesterol (unpublished data). Perifosine was used in liposomal formulations as a promising candidate for tumor treatment [14,15], which, similarly to other alkyl phospholipids (APLs) (Figure 1), easily incorporates into cell membranes in substantial amounts and distributes among intracellular membrane compartments, where it accumulates and interferes with a wide variety of key enzymes [16,17]. APLs are metabolically stable analogues of lysophosphatidyl choline (LPC) and are being developed as anticancer drugs, already in phase III trials [18]. Administration of free (micellar) APLs results in unwanted side effects, reflected in gastrointestinal toxicity and hemolytic activity, which limits the application of higher doses of APLs. To achieve better therapeutic effects of APLs in vivo with fewer side effects, different liposomal formulations of APLs have been tested and showed diminished hemolytic and cytotoxic activity [19]. Molecular structure of APLs closely resembles the structure of lysolipids, which were already used in design of thermo-sensitive liposomes for local release of entrapped drugs by mildly heating affected tissue, where release should take place [20]. However, a proper combination of lysolipids and other lipids resulting in a temperature dependent, burst-like release of majority of liposome contents, which holds great prospects for application in tumor therapy, was achieved only recently [21].

### Perifosine liposomal formulations, micelles, and properties of lysolipids

Alkyl phospholipids are amphiphilic, lysolipid-like molecules and usually form micelles under physiological conditions. However different liposomal formulations of alkyl phospholipids were successfully prepared [22][25]. This is possible only in the presence of lipids or other amphiphiles with complementary molecular shape. Usually cholesterol fulfills this role and enables the preparation of stable liposomal formulations from alkyl phospholipids and lipids of different chain length and head groups [24]. Among different alkyl phospholipids, most investigations with liposomal formulations were performed with perifosine. One of key properties of perifosine containing liposomal formulations, which is unusual for liposomes composed of bilayer forming lipids, is presence of high amounts of micelles in liposomal samples [26,27]. The content of micelles in perifosine liposomal formulations decreases with increasing cholesterol concentration, disappearing roughly at below 1:1 ratio of perifosine to cholesterol [26,28], similarly as capacity of perifosine liposomal formulations for transendothelial delivery. Presence of micelles in perifosine liposomal formulations could be expected, taking into account lipid monolayer experiments, which showed that alkyl phospholipids, below the critical micellar concentration (CMC), insert progressively into lipid monolayers as monomers from the aqueous medium, but above CMC, not only monomers but also groups of monomers (micelles) are transferred into the monolayers [29]. It was also shown that while alkyl phospholipid HePC is miscible with POPC, there is high affinity between HePC and sterols (ergosterol, and cholesterol) and that maximum condensation is reached at a ratio of HePC/sterol around 1:1 (mol/mol) [29]. This kind of behavior is generally known as the condensing effect of cholesterol towards phospholipids [30,31]. Micelles constitute a reservoir of monomers both for monomer insertion between condensed phospholipids and for groups of monomer insertion between fluid phospholipids. Since biological membranes are composed of dynamically condensed domains surrounded by fluid domains, it has been suggested that, above the CMC, alkyl phospholipids can insert into both kinds of phases: as monomers into the condensed phase and as a group of monomers into the fluid phase [32]. This is also in agreement with fluorescence microspectroscopy data, which show that lipophilic phospholipid fluorescent probe NBD-PC, where 7-nitrobenz-2-oxa-1,3-diazol-4-yl (NBD) is attached to a phosphatidylcholine phospholipid is immediately transferred into cells after addition of liposomes with higher than 1:1 ratio of perifosine to cholesterol, whereas liposomes with lower ratio do not interact with cells [26,33]. With electron paramagnetic resonance spectroscopy it was similarly shown that hydrophilic spin probe encapsulated in liposomes with lower than 1:1 ratio of perifosine to cholesterol does not enter cells, whereas starts entering cells immediately after incubation when it is encapsulated in liposomes with higher than 1:1 ratio of perifosine to cholesterol [34]. All of the above results can be explained with the existence of highly mobile phase of free and micellar phase of perifosine in liposomal formulations, which can interact with cells.

### Lysolipids influence endothelial integrity

Lysophosphatidyl choline (LPC) is known to increase endothelial permeability directly [35], it appears to be a proinflamatory mediator, involved in disrupting endothelial barrier function resulting in inflammatory responses in vessel wall [36]. Proinflammatory mediators bind endothelial cell surface receptors and activate signaling cues that induce endothelial cell contraction, resulting in formation of gaps between endothelial cells, which is a primary cause of increased endothelial permeability [37,38]. Cell contraction is achieved by formation of stress fibers, bundles of polymerized actin and myosin filaments. These fibers were shown to be formed in response to many permeability increasing mediators by a monomeric GTPase, RhoA, which plays a central role in increasing endothelial permeability [37]. When the choline group is removed from LPC, lysophosphatydic acid (LPA) is produced, which is also an intercellular signaling molecule influencing target cells by acting on a specific cell-surface receptor [39], activating the RhoA [40,41], and induces prolonged endothelial barrier dysfunction accompanied by a reorganization of the F-actin cytoskeleton [42]. This indicates that choline group doesnt play a significant role in inducing endothelial permeability. However, LPC is quickly metabolized by lysophospholypase and LPC-acyltransferase, and therefore cannot be efficiently used as a medical drug [43]. Therefore alkylphospholipids (APL, also reffered to as alkyl-lysophospholipids) were synthesized by replacing the acyl group of lysophosphatidylcholine (LPC) with an alkyl group. We hypothesize that perifosine, as an LPC analog, which also consists of an 18-carbon alkyl chain and a phosphate group is also capable of inducing endothelial permeability in a similar manner as LPC and LPA. It has been shown that perifosine retains some physiological effects of LPC, for example, both LPC [44,45] and perifosine [46] increase cytosolic Ca^2+^.

### The nonsignificant role of a helper lipid DOPE

Although a hexagonal phase promoting lipid dioleoyl phosphatidylethanolamine (DOPE) [47,48], which is known to be capable of destabilizing endosomal membrane [49][51], was also used in liposomal formulations in combination with perifosine in order to facilitate transendothelial delivery of liposome encapsulated calcein, no benefit in transendothelial drug delivery could be attributed to DOPE. Increased transendothelial delivery was observed for liposomes containing both DOPE and perifosine as well as for liposomes containing only perifosine [6]. This suggests that either perifosine influences destabilization of endosomal memebranes to a much greater extent than DOPE or that endocytosis with destabilization of endosomal membrane might not be the primary mode of transendothelial delivery.

### Physiological conditions - the role of albumin

Designing an efficient transendothelial drug delivery vector, one has to take into account the role of plasma albumin. As it was shown by Huang et al. [35] that, extracellular application of LPC, which exceeded the binding capacity of albumin, activated RhoA and impaired endothelial integrity. The presence of albumin in the medium has the effect of increasing the CMC value by binding lipid molecules and, hence, reducing the concentration of free monomers in the medium [52]. We assume that the role of liposomes is similar to the role of albumin, which acts as a reservoir, gradually releasing albumin bound molecules.

Physiological concentration of albumin ranges from 3.5 to 5g/dl [53], which corresponds to concentration approximately 500 to 750M. Long chain fatty acids, with acyl chains from 16 to 20 carbon atoms and up to 4 double bonds, bind to albumin at 2 strong and 4 weak binding sites [54]. This results in 1 to 1.5mM of strong binding sites on albumin for fatty acid like molecules. Strong fatty acids binding sites of albumin are usually occupied, since free fatty acids (FFAs) are one of the most important metabolites transported by plasma albumin. Although the plasma FFA concentration is quite variable, the molar ratio of FFA to albumin in the plasma is usually in the range from 0.5 to 2, rarely exceeding 3 [55]. Albumin inhibits cytotoxic activity of lysophosphatidyl choline (LPC) by direct binding of LPC [52]. Extracellular application of LPC, which exceeds the binding capacity of albumin, was capable of impairing endothelial barrier function [35]. Under physiological conditions albumin probably binds most of LPC, since its plasma concentration in normal men was found to be around 130M [56]. Since perifosine liposomal formulations contain perifosine micelles in equilibrium with free perifosine, which exhibits critical micellar concentration (CMC) around 0.5mM (unpublished data), we expect that significant amount of perifosine remains unbound under physiological conditions and might be capable of compromising endothelial integrity.

In this article we propose underlying molecular mechanism, which should be taken into account in designing lysolipid containing liposomes as efficient transendothelial delivery vectors in general.

## Presentation of the hypothesis

**Figure 2:**
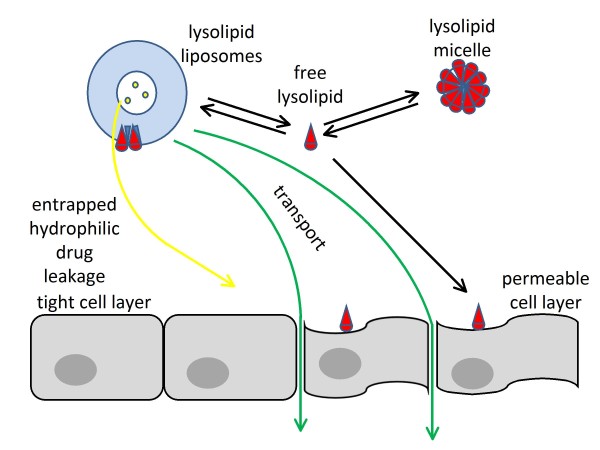
**Increased endothelial permeability is hypothesized to be a consequence of free perifosine action on endothelial cells.** Black arrows indicate a dynamic equilibrium among free perifosine, micelles, and liposomes, all of which constitute a liposomal formulation.

Based on the observation by Orthmann et al. [6], that, in serum free experiments, perifosine containing liposomal formulations increase endothelial barrier permeability, we hypothesize that increased permeability is caused by free perifosine, which induces changes in endothelial cell shape resulting in gaps between endothelial cells, allowing passage of liposomes or their released contents through the compromised endothelial barrier. Lysolipids in a liposomal formulation are in dynamic equilibrium, distributed among liposomes, micelles, and free form (Figure 2). Liposomal formulations therefore represent a reservoir, releasing free lysolipids, which acts on endothelial barrier and locally increase its permeability for drug loaded liposomes.

We hypothesize that lysolipid containing liposomes could be used for transendothelial drug delivery in general due to their several unique properties:

1) Just as any other liposomal formulations they can be used as carriers of encapsulated hydrophilic drugs as well as lipophilic compounds;

2) They can release lysolipids in a controlled manner by varying lipid composition of liposomal formulation. The highest concentration of released lysolipids, which can compromise endothelial integrity, is therefore always in vicinity of liposomes with encapsulated active drug;

3) Leakage of liposome encapsulated drug, due to liposome destabilization, which is caused by lysolipid depletion, is greatest at the site of interaction of free lysolipids with endothelial barrier;

4) Incorporation of superparamagnetic iron oxide nanoparticles in lysolipid containing liposomes could help in achieving high concentration of the liposomes at a particular point of the body using external magnetic field;

5) Since lysolipid containing liposomes are thermosensitive, the release of the liposome contents can be further accelerated by heating the local tissue.

Free lysolipids from liposomal formulations might also induce transendothelial transport *in viv*o in a similar way as in serum free conditions, provided that free perifosine concentration is high enough to saturate binding sites on albumin.

## Testing of the hypothesis

This hypothesis could be verified by measuring under *in vitro* conditions:

· Binding of perifosine and other lysolipids to lysophospholipid receptor (LPL-R) group;

· Formation of stress fibers and subsequent cell contraction;

· Possible activation of RhoA, and endothelial barrier dysfunction by measuring transendothelial resistance (TEER), before, during, and after the incubation of liposomal formulations with endothelial cell layer;

· Leakage of liposome contents with respect to lysolipid depletion from liposomes as a function of other lipid components of liposomal formulation.

The hypothesis of *in vivo* nonspecific transendothelial vector design could be verified by measuring:

· Influence of free lysolipids in presence of albumin, at higher or lower than albumin concentrations, on endothelial permeability;

· Binding constant and number of binding sites for the lysolipid binding to albumin in comparison to LPC binding;

· Extent of transendothelial delivery of liposome encapsulated marker by liposomal formulations made of lysolipids with different CMCs in comparison to perifosine and LPC, in presence and absence of albumin;

· Critical micellar concentration (CMC) of perifosine as compared to other lysolipids and whether transendothelial delivery of liposome encapsulated marker depends on CMC of a lipid used as a component of the liposomal formulation;

· Appearance of pathogens at the basolateral side.

## Implications of the hypothesis

If the hypothesis were true, lysolipid containing liposomal formulations might be used as endothelial transport vector, where free lysolipids, as a component of liposomal formulation, would locally compromise endothelial integrity in a similar manner as lysophosphatidyl choline, thus increasing the amount of delivered therapeutics into a diseased tissue.

## Misc

This work was supported in part by Slovenian Research Agency Program P1-0060 Experimental Biophysics of Complex Systems, and in part by Center of Excellence Namaste.

## Competing interests

The authors declare that they have no competing interests.

## Authors contributions

TK contributed to conception and design of the hypothesis, interpretation of previously published data, and has been involved in drafting the manuscript. JS gave the final approval of the version to be published. All authors read and approved the final manuscript.
